# Review of leptospirosis in dogs from Mexico: Epidemiology, diagnosis, prevention, and treatment

**DOI:** 10.14202/vetworld.2024.1356-1361

**Published:** 2024-06-21

**Authors:** Estefanía Andrade-Silveira, Antonio Ortega-Pacheco, Matilde Jiménez-Coello, María Cárdenas-Marrufo

**Affiliations:** 1 Department of Animal Health and Preventive Medicine, Autonomous University of Yucatán, Mérida, Yucatán, México; 2 Microbiology Laboratory, CIR “Hideyo Noguchi”, Autonomous University of Yucatán, Mérida, Yucatán, México; 3 Interinstitutional Clinical and Epidemiological Research Unit, Autonomous University of Yucatan, Merida, Yucatan, México

**Keywords:** dog, *Leptospira*, leptospirosis, Mexico and diagnosis, prevalence, prevention, treatment, vaccine

## Abstract

Leptospirosis, classified by the World Health Organization as an emerging and neglected disease, is caused by the zoonotic pathogen *Leptospira interrogans*. This review aims to outline the Mexican epidemic of *L. interrogans* in dogs, including diagnosis and prevention methods. This review article searched articles from the publishers Wiley, Springer, PubMed, Redalyc, SciElo, and Elsevier. Among the 200 Mexican articles concerning Leptospira epidemiology, diagnosis, treatment, and vaccination, those that failed to meet the set inclusion criteria were excluded. The worldwide study of *L. interrogans* has focused on this bacterium. In Mexico, up-to-date information on canine prevalence, diagnosis, and vaccine use is scarce. Flow cytometrically detected Salmonella serovars differ from those in current vaccines, emphasizing the importance of broadening vaccine serovar coverage.

## Introduction

Leptospirosis is a bacterial disease that infects both animals and humans. More than one million people can be affected by *Leptospira*, causing at least 60,000 deaths/year with a death rate of 20% [1]. The World Health Organization considers *Leptospira* a significant public health risk in human and veterinary medicine due to its increasing prevalence as a zoonotic disease. Ten out of every 100,000 individuals can contract leptospirosis in tropical areas [2, 3]. Leptospirosis affects the most vulnerable populations in rural and urban environments [4]. Direct contact with urine from an infected person or contaminated water serves as the primary cause of infection. Serovar Canicola resides in dogs as a maintenance host. Asymptomatic kidney carriers, known as maintenance hosts, excrete leptospiras in their urine, whereas accidental hosts are individuals who come into contact with infected urine. For years, dogs can harbor and excrete various pathogenic serovars of this bacterium in their urine [5–7]. *Leptospiras* reside in the renal tubules of asymptomatic animals, such as dogs, cows, pigs, horses, cats, rodents, and opossums [8, 9]. 35 *Leptospira* species, organized into three phylogenetic groups, reflect varying bacterial virulence [10]. Dogs’ vaccines cover 4–6 cross-agglutinating serovars. However, since 1960, attempts have been made to achieve this cross-protection against the different serogroups, but they have not been successful [11].

Leptospirosis, with similar clinical symptoms to rickettsiosis [12], can be difficult to distinguish from other diseases. Dogs in recovery from leptospirosis pose a risk for zoonotic transmission due to their prolonged shedding of the bacteria as asymptomatic carriers [13]. *Leptospira* can survive in freshwater, moist alkaline soils, vegetation, and mud [7]. Age, breed, sex, environmental conditions, rainy seasons, and environmental temperature pose risks for dogs [9]. Dogs living in patios, with water tanks present and poor sanitary conditions, carry distinct risk factors [9]. Rodents carry *Leptospira* bacteria and their presence and unsealed food storage for dogs contribute to infection [14].

Leptospirosis remains endemic in both canine and human populations in Mexico. In 1920, Noguchi and Kliger identified the endemic bacterium causing the disease in Mérida, Yucatán [4, 9]. Yucatán, a state from southern Mexico, has presented ideal conditions for the transmission of *Leptospira*, due to its climate, temperature, and humidity [4]. In Mexico, leptospirosis is a notifiable disease according to the Official Mexican Standard for the Epidemiological Surveillance, Prevention, and Control of Leptospirosis in Humans, NOM-029-SSA2-1999 [4]. Since 2000, Sinaloa state has the highest number of cases of *Leptospira* in people nationwide; from 2005 to 2016, about 297 cases of *Leptospira* have been reported in humans [4]. This review describes the epidemiology, diagnosis, and prevention of leptospirosis in dogs in Mexico.

## Review Methodology

Systematic review was carried out using databases, such as Wiley, Springer, PubMed, Redalyc, SciElo, and Elsevier. Articles on this topic were located using keywords such as Vaccine, Leptospira, Dog, Treatment, Prevention, Mexico, Diagnosis, Prevalence, and Leptospirosis. The articles selected for review were published from 2000 to 2023, in English or Spanish, by the specified publishers, and their topics included *Leptospira* in dogs, *Leptospira* in Mexico, epidemiology, diagnosis, vaccination, and treatment. 43 articles were chosen from the initial 200, after reviewing their titles and abstracts according to the inclusion criteria.

## Epidemiological Situation in Dogs in Mexico

**Figure-1:**
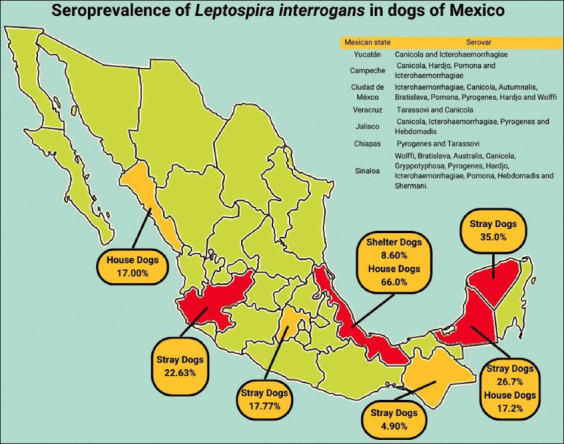
Seroprevalence of *Leptospira interrogans* in dogs with owners, stray dogs, and from shelters in the different states of Mexico. [1, 15].

The prevalence in Mexico differs from one state to another within the Mexican Republic. The prevalence of *Leptospira* varies among Mexico’s states, as shown in Figure-1 [1, 15]. Close relationships between humans and domesticated dogs in both rural and urban areas of Mexico result in epidemic outbreaks, with stray dogs presenting a significant risk to domesticated dogs [8, 14].

In 2008, a 35% seroprevalence of Canicola and Icterohaemorrhagiae in 400 stray dogs in Mérida, Yucatán, was discovered post-hurricane season [9, 16]. In Campeche’s neighboring state, strays and domiciled dogs yielded seroprevalences of 17.2% and 26.7%, respectively, with most dogs testing positive for Canicola, Hardjo, Icterohaemorrhagiae, and Pomona serovars [17]. 36 community members and their 29 dogs were found to have the Tarassovi serovar in 66% of the cases. Dog owners possess *Leptospira interrogans* antibodies due to inhabiting the same polluted environment [2, 7]. In Veracruz dog shelters, Canicola serovars infected dogs despite vaccination due to their exposure to highly contaminated environments [18].

Stray dogs within the state of Chiapas reported a prevalence of 4.9%, concluding that stray dogs are an important reservoir of *L. interrogans* in the city [19]. 17% of the 116 dogs in Culiacan, Sinaloa, were reported to carry Wolffi, Bratislava, Australis, Canicola, Grippotyphosa, Pyrogenes, Hardjo, Icterohaemorrhagiae, Pomona, Hebdomadis, and Shermani [9]. In Mexico City, a seroprevalence of 17.77% was obtained in a population of 45 stray dogs, showing titer against the serovars Icterohaemorrhagiae, Canicola, Autumnalis, Bratislava, Pomona, Pyrogenes, Hardjo, and Wolffi [20].

## Diagnosis

The microagglutination test (MAT) and enzyme-linked immunosorbent assay (ELISA) tests are the gold-standard tests for *Leptospira* diagnosis in dogs in Mexico [21]. The ELISA test, which is simple, convenient, and secure, operates without utilizing live Leptospiras. Depending on the ELISA variant, it identifies immunoglobulin M (IgM) or IgG antibodies [13]. An early, accurate diagnosis saves lives. The success of the ELISA test depends on the stage of the immune response; *Leptospira* IgM antibodies can be detected as soon as 4–7 days after infection; the day of infection is difficult to estimate, so the onset of clinical signs is considered [22].

The MAT test distinguishes various *Leptospira* serovars for diagnosis. In Mexico, the use of serovars Wolffi, Bratislava, Australis, Canicola, Grippotyphosa, Pyrogenes, Hardjo, Icterohaemorrhagiae, Pomona, Hebdomadis, and Tarassovi has been reported [7, 21]. Because the nature of the MAT test does not provide reliable data in acute leptospirosis cases in humans and dogs, new diagnostic alternatives have been implemented, such as real-time polymerase chain reaction (PCR) which indicates a recent infection, in which vaccination does not interfere with the diagnosis of acute infection; however, its use is more limited because of its cost and need of special equipment [7, 23].

A high titer in the MAT test may make it challenging to distinguish between chronic infections, previous exposures, and acute infections [24]. To observe the clumps in the test, a special dark-field microscope is required. To maintain agglutination, this technique necessitates staff with expertise. This test is known for detecting serovar agglutinating antibodies, making it quantitative in nature. The test result is expressed in dilution titers that can vary from 1:20 to 1:20,480 [12]. The agglutination of live serovars in serial dilutions of patient serum forms the basis for MAT diagnosis [22]. Vaccines can show titers of 1:100–1:200 on the MAT test during weeks 12–16 after vaccination; however, titers of 1:800 in unvaccinated dogs can suggest a presumptive diagnosis of active infection; however, recently vaccinated dogs can also reach titers of 1:800, making it difficult to obtain a diagnosis of the disease using the MAT test [25].

## Vaccination

At present, there are few reports on vaccination against *Leptospira* in Mexico; on the Mexican market, there were a total of 26 biological products manufactured by 15 different laboratories; 19 of the vaccines contain only the serovars Canicola and Icterohaemorrhagiae; and seven vaccines contain the serovars Canicola, Icterohaemorrhagiae, Grippotyphosa, and Pomona [26]. The vaccine should be administered at 8 and 9 weeks of age with subsequent doses at 15 days and 6 months, followed by an annual application. In North America, leptospirosis caused by the serovars Canicola, Grippotyphosa, Icterohaemorrhagiae, and Pomona can infect vaccinated dogs. Although lethality is reduced, leptospirosis can still develop in vaccinated dogs with high bacterial exposure [27, 28].

Vaccines against *L. interrogans* can prevent clinical disease and mortality but do not hinder renal excretion [29, 30]. In Mexico, the *Leptospira* vaccines consist of either bacterins or antigens derived from bacterial cell walls, the majority of which cover the serovars Icterohaemorrhagiae, Canicola, Pomona, and Grippotyphosa [26, 31]. According to Table-1, these vaccines must be regularly boosted to preserve immunity against specific serovars. Every year, during MAT testing, this phenomenon has been observed. After receiving the vaccine, the dogs no longer have antibody titers [26].

**Table-1:** Commercial vaccines frequently used in Mexico and the serovars present in these vaccines.

Laboratory	Commercial name	Serovar
Virbac	Canigen MHA2PPi/L	Canicola and Icterohaemorrhagiae
Zoetis	Vanguard plus 5/CV-L	Canicola and Icterohaemorrhagiae
Zoetis	Vanguard plus 5/L4/CV	Canicola, Icterohaemorrhagiae, Grippotyphosa, and Pomona
Holland	Canomune puppy dha2ppi +l4	Canicola, Icterohaemorrhagiae, Grippotyphosa, and Pomona
Novibac	DHPPi-RL	Canicola and Icterohaemorrhagiae
Novibac	Nobivac lepto	Canicola and Icterohaemorrhagiae
MSD	Quantum dog da2ppvl+cv	Canicola and Icterohaemorrhagiae
Merial	Recombitek	Canicola and Icterohaemorrhagiae
Bio Zoo	Inmunovax 3 DH-L	Canicola and Icterohaemorrhagiae
Pet’s Pharma	Bioprevent Booster Plus	Canicola and Icterohaemorrhagiae
Chinoin	Vacugen 6L	Grippotyphosa, Canicola, Pomona, Tarassovi, Icterohaemorrhagiae, and Wolffi
Lapisa	Providean Viratec 10	Icterohaemorrhagiae, Canicola, Pomona, and Grippotyphosa

Vaccines against *Leptospira* strains from the Canicola and Icterohaemorrhagiae serogroups have been used for 50 years. Although vaccinated against these bacteria, some dogs have shown clinical signs related to distinct serogroups [32, 33]. Despite vaccines, *Leptospira* infection can lead to fatalities in dogs. Vaccination does not always guarantee complete protection against *Leptospira* serovars, and vaccines are designed to prevent disease, but not infection [1, 34, 35]. The prevalence of leptospirosis in dogs has increased since 1990s due to infections caused by different serovars that were not found in bivalent vaccines [25].

In the state of Mérida, capital city of Yucatán, Mexico, in 2007, a study was carried out on 348 domiciliated dogs and found that 52.4% of them had been vaccinated against *Leptospira* [28]. In 2020, a study carried out in Germany to assess factors associated with vaccination in dogs showed that only 46.8% of them were vaccinated annually, and owners claimed that vaccines were unnecessary and expensive [36]. Among 60% of dogs brought to the United Kingdom veterinary clinics have outdated vaccination records due to owner concerns over side effects, frequent vaccination schedules, and socioeconomic factors [37]. Vaccination shields against infectious diseases that threaten mortality and zoonoses; for leptospirosis, the vaccine lessens kidney disease severity and human transmission, whereas non-compliance with immunization facilitates the disease spread [20, 36, 37].

Inactivated vaccines offer immunity through humoral response to lipopolysaccharide (LPS), as well as passive transfer of anti-LPS antibodies, shielding against specific antigen-carrying serovars [32]. In a 2022 study carried out on 118 dogs, of which 94 were vaccinated and 24 were not, which were monitored with various tests, including MAT, ELISA, and urine PCR, and it was found that with vaccination, it was possible to obtain an IgG answer and partial protection against kidney infection [31]. 46.5% of the 580 dogs with hepatic or renal disease following Canicola and Icterohaemorrhagiae vaccination exhibited hepatitis, whereas 21.6% were diagnosed with acute kidney injury [27].

Annually, revaccinating stimulates IgG and T-cell responses, illustrating efficient and enduring immunological memory [33]. Antibodies against leptospiral LPS provide immunity post-infection, and vaccines similarly induce immunity involving lipopolyoid binders [13]. During weeks 5–27 and 56 after the first vaccination, high protection against both Canicola and Icterohaemorrhagiae infections is induced by the vaccines. However, for optimal cross-protection against leptospirosis, annual revaccination or booster shots are suggested [29, 38].

## Treatment

Antibiotics such as penicillin, amoxicillin, clavulanic acid, cephalexin, ceftriaxone, doxycycline, tetracycline, streptomycin, and enrofloxacin have been reported for the treatment of leptospirosis in dogs from Mexico [14, 20]. Treatment should be initiated at the onset of suspected disease for optimal success, as the disease often fails in severe stages with kidney lesions, despite reports of shorter duration with antibiotic therapy [14, 26]. The therapeutic plan should be based on the clinical assessment and disease severity. The antibiotic administration will depend on the patient’s tolerance to oral medication; it is suggested to start with intravenous antibiotic therapy in the case of gastroenteric symptoms [11].

Penicillin and doxycycline are the initial antibiotics for leptospirosis treatment. Doxycycline should be given in doses of either 5 mg/kg every 12 h or 10 mg/kg every 24 h for 14 days. 12 h apart, 25,000–40,000 U/kg of penicillin, or 8 h apart, 20–30 mg/kg doses of amoxicillin are given intravenously [11, 20]. The recommended doses should be adjusted based on the patient’s renal function [11]. Consider potential side effects such as vomiting and esophageal irritation during doxycycline treatment. 10 mg/kg of enrofloxacin every 24 h for 10 days has been proven to be equally effective as doxycycline [20]. There is recorded resistance for sulfonamide, neomycin, actidione, polymyxin, vancomycin, and rifampicin against *Leptospira*, whereas resistance for doxycycline and penicillin remains unknown. Antimicrobial resistance in leptospirosis is not a significant issue [39].

## Discussion

Since dogs are closely related to humans, *L. interrogans* is a significant bacterium to consider in the differential diagnosis of diseases due to the potential for early detection and prevention of mortality. In Mexico, leptospirosis is one of the notifiable diseases based on the Official Mexican Standard for the Surveillance, Prevention, and Epidemiological Control of leptospirosis in humans, NOM-029-SSA2-1999 [4]. In areas with stray dogs and rodents, leptospirosis, a neglected disease, affects vulnerable rural and urban populations [4, 26]. The place of residence of dogs is an important risk factor; work shows that dogs that go outside are prone to having *Leptospira*, unlike dogs that do not go outside [9]. The dearth of adequate waste management and urban planning in urban areas is predicted to contribute to a surge in cases due to weather events augmented by climate change [40].

In Mexico, stray dogs carry and spread this bacteria, posing a risk to both canine and human populations [9, 26]. Due to their sniffing, licking, and courtship behaviors, stray dogs can potentially transmit infections to both other dogs and people, making them a concern for the health of domiciliated dogs [26]. At the Mexican City canine control center, clinically healthy dogs with high titers were found, suggesting that they unknowingly harbored pathogenic leptospires capable of environmental transmission [41]. Since cats are significant carriers, who can shed leptospires in their urine for as long as 3 months, stray or domestic dogs near them are more prone to testing positive [4]. In the city of Mérida, capital city of Yucatán, in 2020, an epidemiological study was carried out on 260 cats domiciled where a seroprevalence of 17.7% of the different serovars of *L. interrogans* was found, including Australis, Pyrogenes, Grippotyphosa, Bratislava, Canicola, and Icterohaemorrhagiae [42].

In Mexico, MAT is the most commonly utilized test for *L. interrogans* diagnosis in dogs. Although it is considered the gold-standard test for the diagnosis of *L. interrogans*, it requires specialized training, which makes access difficult for veterinary clinicians. The MAT test cannot distinguish vaccinated from non-vaccinated dogs, necessitating an additional test for validation [24]. Instead, pursue alternative diagnostic techniques that offer faster results. It is vital for dog owners to understand their pets’ risks around other animals and practice good hygiene to prevent contagion.

Dogs should be vaccinated against *L. interrogans* yearly to prevent the disease. While vaccinated, dogs can contract diseases from serovars excluded from vaccines, such as Grippotyphosa, Pomona, Bratislava, Australis, Copenhageni, and Icterohaemorrhagiae [32, 33, 43]. In Mexico, there are at least 26 vaccines that protect against different serovars; most vaccines have protection against the Canicola and Icterohaemorrhagiae serovars, while only a few add Grippotyphosa and Pomona in the biological [26].

The epidemiological studies carried out to date have reported the presence of other pathological serovars circulating in canine populations such as serovars Hardjo, Tarassovi, Pyrogenes, Bratislava, Australis, Wolffi, Hebdomadis, and Shermani, which are not included in commercial vaccines. Some serovars share the same antigens, which cause a slight cross-reaction, such as Australis and Bratislava [25]. In Mérida, Mexico, only 53% of owners vaccinate their dogs with the multiple vaccines that include *L. interrogans*, leaving 47% of dogs exposed to serovars found in the environment [28]. 46.8% of domiciled dogs in Germany are annually revaccinated [17]. Socioeconomic factors and fears may underlie the reason for some people’s reluctance to get vaccinated [11].

## Conclusion

In Mexico, the prevalence, diagnosis, and prevention of *L. interrogans* in dogs remain poorly documented. Despite its presence in multiple Mexican states, little is known about the distribution and pathogenic serovars of an endemic bacterium in Mexico, which can impact human and animal health. Precise prevention measures require knowledge of the distribution and presence of pathogenic *Leptospira* serovars, as humans are at risk due to their proximity to infected dogs. Commercial vaccines for *Leptospira* interference in Mexican dogs cover only a limited range (2–4) of the prevalent serovars. To determine the circulating serovars and expand vaccine coverage, it is recommended to conduct more epidemiological studies.

## Authors’ Contributions

All authors contributed to the conception and design of the review article. EA: Conducted the collection of information, the analysis of the articles, and the writing of the article. AO, MJ, and MC: Reviewed the writing and provided comments to improve the article. All authors have read, reviewed, and approved the final manuscript.
